# In-situ detection of delamination reinitiation in carbon fiber reinforced polymers post barely visible impact damage

**DOI:** 10.1016/j.heliyon.2024.e37782

**Published:** 2024-09-12

**Authors:** Kais Jribi, Boutros Azizi, Alberto W. Mello

**Affiliations:** a Aerospace Engineering Department, Embry-Riddle Aeronautical University, Daytona Beach, FL, 32114, USA; b Engineering Fundamentals Department, Embry-Riddle Aeronautical University, Daytona Beach, FL, 32114, USA

**Keywords:** Barely visible impact damage, Compression after impact, Digital image correlation, Delamination propagation

## Abstract

In this study, advancements are presented in the in-situ detection of delamination reinitiation from Barely Visible Impact Damage (BVID) in composite materials, utilizing enhancements in Digital Image Correlation (DIC) techniques during a Compression After Impact (CAI) test. The study measured strain fields in the longitudinal, transverse, and shear directions, focusing specifically on the point of highest out-of-plane displacement to identify the onset of delamination propagation from BVID sites generated at different impact energy levels. By correlating the measured strains with the peak out-of-plane displacement, a unique determination of onset damage reinitiation associated with BVID during CAI testing was achieved. This method introduces a refined in-situ assessment technique for structural integrity, allowing for the early detection of critical damage propagation in composite materials.

## 1 Introduction

Carbon Fiber Reinforced Polymers (CFRPs) have become a cornerstone in the aerospace industry, lauded for their superior stiffness and strength-to-weight ratios compared to traditional metallic alloys. The ability to design complex sections with fewer individual parts has not only streamlined manufacturing processes but has also mitigated the risk of component failure due to faulty machining or assembly [1]. However, the inherent anisotropic nature of CFRPs presents unique challenges, notably their vulnerability to low-velocity impacts, often leading to barely visible impact damage (BVID) [[2], [3], [4]].

**Fig. 1:**
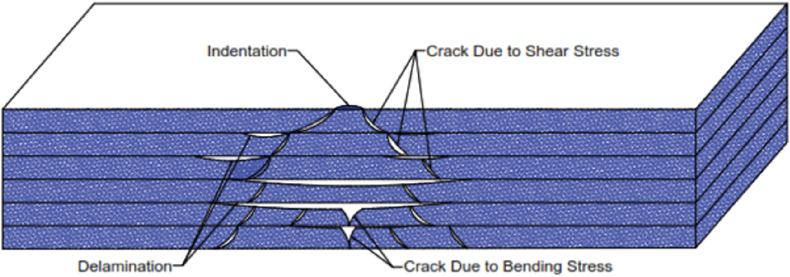
Schematics of the basic anatomy of a BVID [4].

BVID is a form of internal damage that manifests as cracks, delamination, or fiber breakage, often without leaving noticeable external markings [2,4]. This insidious damage is a significant concern as it can act as a nucleation site for further degradation under cyclic loading or stress concentrations, potentially leading to catastrophic failure if not detected and addressed [3]. The schematic representation in Fig. 1 illustrates the typical anatomy of a BVID [4], highlighting the hidden nature of the damage and the complexity of its detection.

### 1.1 Classification of damage in aerospace composites

The aerospace industry meticulously classifies composite damage based on its severity to guide appropriate action [3]. The most subtle form, barely visible damage, is the most challenging to detect and often overlooked, yet it can be the precursor to catastrophic failures. As the damage progresses, it becomes more detectable but may still be tolerated within specific limits, known as the damage tolerance domain. Beyond this threshold, repairs become necessary as the residual strength of the structure diminishes. The most severe damage category involves substantial structural compromise, rendering the component unsuitable for use.

Impact events, encompassing phenomena like hail damage, tool drops, bird strikes, and debris encounters, account for approximately 80 % of structural damage in composite materials [2,5]. Among these, low-velocity impacts pose a particular challenge as they frequently induce BVID, which can severely degrade the component's residual strength [6]. A study on the residual strength assessment of composite samples subjected to low-velocity impacts under compressive stress revealed a substantial decrease of up to 60 % of pre-damage strength due to local buckling caused by delamination [[6], [7], [8], [9], [10]]. This underscores the importance of continuous monitoring and assessment of BVID to prevent its progression and avert catastrophic failures.

### 1.2 Unveiling BVID in CFRPs

The inherent resistance of CFRPs to permanent deformation often makes BVID imperceptible to the naked eye, posing a significant challenge for detection [11]. BVID failure mechanisms, primarily comprising delamination and cracks induced by shear and bending stresses, are influenced by various factors, including material properties, impact characteristics, boundary conditions, and impactor geometry [4,12,13].

The presence of BVID can drastically compromise the structural strength of a composite component. While delamination may not propagate significantly under tensile loads, compressive loads can trigger its expansion across plies with different fiber orientations, ultimately leading to a substantial reduction in load-bearing capacity [[6], [7], [8], [9], [10],^14^]. This phenomenon underscores the need for robust methodologies to predict and analyze delamination growth under diverse loading scenarios.

Detection and assessment of BVID typically require the use of advanced non-destructive evaluation (NDE) techniques, such as ultrasonic scans [15,16], X-ray computed tomography (CT) [17,18], or thermographic inspections [19,20]. These methods can identify and characterize the extent of internal damage with significant limitations. Ultrasonic inspection requires a coupling medium to facilitate sound wave transmission and the expertise to set up and interpret the results. On the other hand, X-ray inspections are constrained by strict safety protocols, making them unfavorable for periodic inspections. The thermographic inspection also has a few downfalls, due to environmental conditions and surface emissivity variability.

With that in mind, various attempts were made to detect the delamination reinitiation using Digital Image Correlation (DIC) techniques. These approaches, as promising as they might be, fail to provide definitive results indicating the threshold at which the onset of the delamination reinitiates [21,22].

### 1.3 Advancements in composite damage tolerance research

Research efforts in composite damage tolerance have focused on understanding the behavior of BVID and subsequent delamination under different loading conditions. Advanced techniques such as three-dimensional finite element analysis have been employed to monitor and quantify strain energy release during delamination progression [23]. Ultrasonic scanning has been utilized to visualize and quantify stiffness reduction across delaminated areas, revealing the material's degradation pattern under stress [24]. Moreover, studies have investigated the response of composites to cyclic compressive loads after impact, demonstrating a significant decrease in load-bearing capacity [25]. Numerical models have been developed to predict the buckling response and delamination propagation pathways, aiding in the failure analysis of composites [26]. High-fidelity finite element models, coupled with experimental quasi-static indentation tests, have enabled researchers to trace the evolution of damage from initial impact to subsequent loading [27].

Several methodologies have been proposed to predict and analyze delamination growth, including stress-strain based models, fracture mechanics based models, cohesive-zone models, and extended finite element-based models [28]. However, these methods face limitations in capturing the complex nature of damage progression, the ambiguity in damage characterization, and the heterogeneity of composite structures [28].

With that in mind, various attempts were made to detect the delamination reinitiation using Digital Image Correlation (DIC) techniques. These approaches, as promising as they might be, fail to provide definitive results indicating the threshold at which the onset of the delamination reinitiates [21,22].

### 1.4 Digital Image Correlation (DIC) in damage assessment

Digital Image Correlation (DIC), as a non-contact optical technique, has emerged as a valuable tool for damage assessment in composite materials. It offers full-field measurements of displacements and strains, providing valuable insights into the structural response and damage evolution under various loading conditions [29]. The evolution of DIC technology, particularly the development of 3D DIC systems with multiple cameras, has enabled the accurate capture of complex, three-dimensional deformations, enhancing the sensitivity and accuracy of BVID detection [[30], [31], [32]].

Advancements in DIC algorithms, such as the inverse compositional Gauss-Newton (IC-GN) method with second-order shape functions, have further improved the accuracy and efficiency of measurements, especially for non-uniform deformations [33]. Moreover, the use of synthetic speckle patterns for calibration has enhanced the robustness and precision of stereo-DIC systems [34].

In the context of SHM, DIC has been used to investigate the impact behavior of various composite materials, including hybrid composites with basalt and carbon fibers [16]. DIC measurements have also been employed to validate numerical models that predict damage initiation and propagation in composite structures [4]. Multi-camera DIC systems have been implemented in industrial settings for non-destructive testing of large-scale structures like bridges and wind turbine blades [35,36].

### 1.5 Research objectives and significance

This research aims to address the limitations of current BVID detection methodologies by developing a novel approach based on DIC. The primary objective is to establish a correlation between surface strains and out-of-plane displacements, which can be readily measured using DIC, to accurately identify the threshold at which delamination initiates from BVID. By utilizing a combination of experimental testing and numerical simulations, this study will investigate the complex relationship between surface strain patterns and the onset of delamination propagation under various loading conditions.

The proposed approach will be validated through a series of experiments on CFRP laminates with different layup configurations and subjected to varying levels of low-velocity impact energy. The results obtained from DIC measurements will be compared with traditional methods, such as ultrasonic testing and X-ray tomography, to assess the accuracy and effectiveness of the proposed methodology. The findings of this research will contribute to the development of more reliable and efficient SHM techniques for composite structures, ultimately leading to improved safety and performance in aerospace and other critical applications.

### 1.6 Delamination reinitiation: a critical focus

A crucial aspect of BVID assessment is understanding the conditions under which delamination, a common form of damage in composites, reinitiates and propagates. This phenomenon is particularly relevant in scenarios where composite structures experience cyclic loading or compressive stresses after an initial impact [9,10]. Previous studies have attempted to detect delamination reinitiation using DIC, but these approaches often lacked definitive criteria for identifying the precise threshold at which delamination begins [9,10].

This research aims to address this gap by developing a novel DIC-based methodology that can accurately estimate the threshold for delamination reinitiation. By analyzing surface strains in the longitudinal, transverse, and shear directions, particularly at the point of maximum out-of-plane displacement, this study seeks to establish a clear correlation between strain patterns and the onset of delamination propagation [4]. This approach offers a more refined and in-situ assessment technique for structural integrity, enabling early detection of critical damage propagation in composite materials.

### 1.7 Experimental setup and methodology

To achieve this goal, a comprehensive experimental setup will be employed, involving the creation of BVID in CFRP specimens through controlled low-velocity impact tests. The specimens will then be subjected to compression after impact (CAI) tests while simultaneously monitoring surface strains using DIC. The data collected from these experiments will be analyzed to identify the critical strain values and displacement thresholds that correspond to the onset of delamination reinitiation.

The experimental setup will include high-speed cameras for capturing DIC images, a universal testing machine for applying compressive loads, and advanced software for data processing and analysis. The specimens will be carefully prepared with speckle patterns to ensure accurate DIC measurements, and the entire experimental process will be meticulously documented to ensure reproducibility and reliability of the results.

### 1.8 Expected outcomes and contributions

Building upon previous research and addressing the limitations encountered in existing methodologies for detecting the reinitiation of delamination from a BVID, this study introduces an innovative strategy for accurately estimating the threshold at which delamination reinitiates. This approach uses DIC techniques to evaluate surface strains as a definitive indicator of delamination onset. This method stands out from earlier attempts, which struggled to define clear boundaries for reinitiation by utilizing precise monitoring and analysis of surface strain patterns. The proposed technique offers a more accurate and dependable means of pinpointing the critical juncture of damage progression.

## 2 Experimental setup and methodology

### 2.1 Inducing BVID for damage analysis

**Fig. 2:**
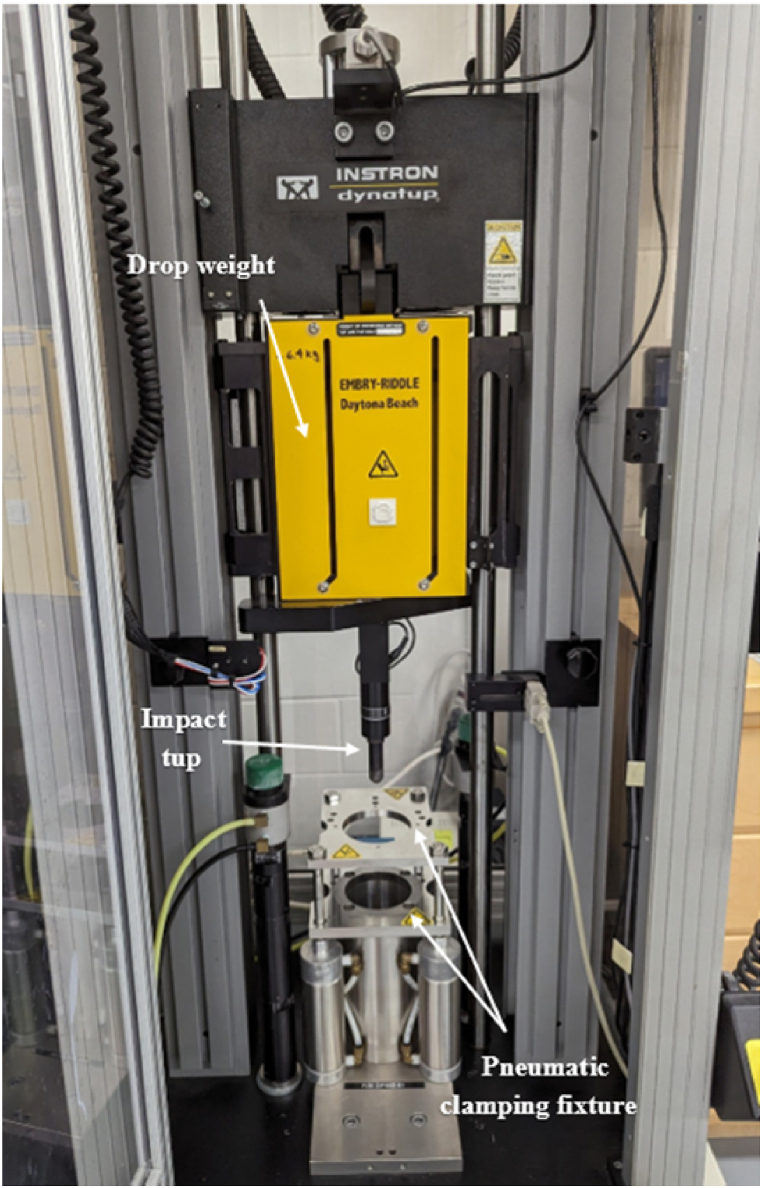
Impact test frame - 9250HV system setup.

A total of 50 coupons with a layup sequence of (45/90/-45/0/-45/0/-45/0/45/90/45/90)s denoted as ‘AS’ and a (45/0/-45/90)3s denoted as ‘S’ passed the quality control which included visual inspection for defects, dimensional verification to ensure compliance with specified tolerances, and non-destructive testing using X-ray scans to detect any internal anomalies or defects. Focusing first on determining the most effective support conditions. Clamped edges were identified as the superior support mechanism, as they prevent the sample from dislodging, thus avoiding excessive damage that could occur with simple support methods [37]. Clamped supports also offer a more realistic damage initiation simulation than back-face support. The current 9250HV system (Fig. 2) employs a pneumatic clamping fixture that applies 448 ± 35 kPa of pressure to ensure the coupons are securely clamped and a rebound stopper to prevent the drop sled from striking the sample more than once.

**Fig. 3:**
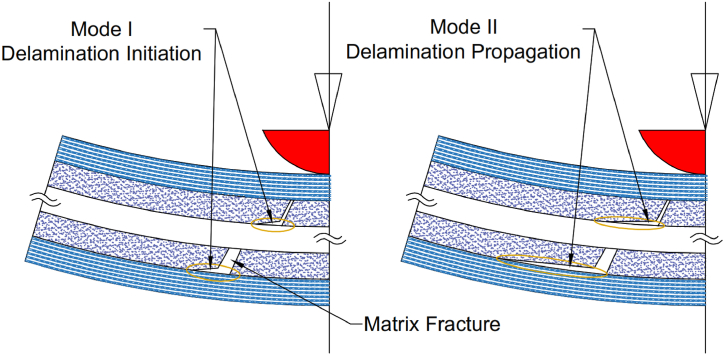
Schematics of delamination initiation (mode I) and propagation (mode II) [4].

The choice of tup diameter and impact energy levels is vital, as the study aims to produce a range of BVID perimeters. Samples were subjected to impacts with energy levels from 2.5 to 15 J using a blunt 12.7 mm diameter hemispherical tup. These energy levels induced matrix cracking and delamination while avoiding fiber breakage and perforation, as seen in Fig. 3 [38].

**Fig. 4:**
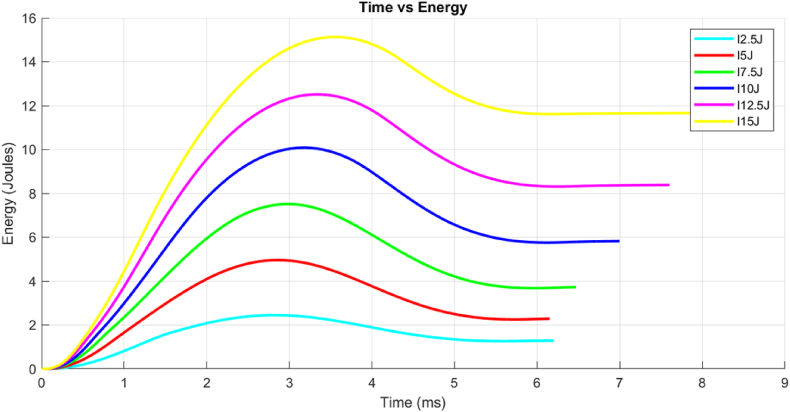
Time (ms) vs. Energy (J) curve at varying energy levels.

Furthermore, the setup included two channels for capturing impulse data from the tup and dynamic load integrated into the data acquisition system. The data acquisition frequency was set to capture data over 100 ms to avoid prematurely ending the recording, providing around 32,000 data points. This setup provided a dataset comprising load, velocity, impact energy, and time, facilitating consistent analysis of impact parameters. Fig. 4 presents the relationship between time and energy values, demonstrating that higher impact energies result in longer rebound times for the sample resulting in more damage.

**Fig. 5:**
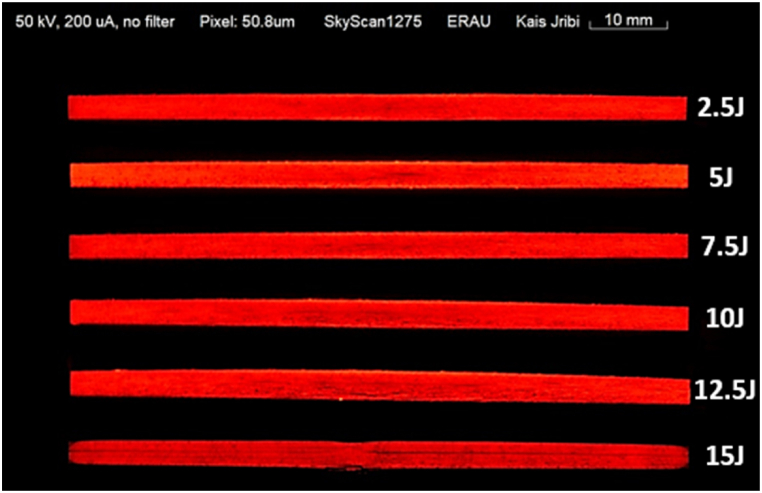
Damage profiles from LVI with different energy levels (from the top: 2.5J, 5J, 7.5J, 10J, 12.5J, and 15J), obtained with Bruker SkyScan 1275 at 50 kV and 200μA.

The samples are then scanned using the Bruker SkyScan 1275 3D X-ray microtomography to detect the damage profiles caused by different energy levels. The voltage and current settings were set to 50 kV and 200μA, respectively. A black body color profile is then applied to generate a more visible damage profile that distinguishes the fiber and epoxy resin variation from actual damage. The findings related to impact damage, illustrated in Fig. 5, reveal the anticipated damage patterns that align with the various energy levels utilized. However, it is essential to acknowledge the challenges involved in accurately gauging the extent of damage within a controlled setting. Firstly, composite material's complex and varied nature can lead to inconsistent responses to impact, creating non-uniform damage distributions that are challenging to measure with precision. Secondly, the current limits in imaging technology may not adequately capture the most diminutive forms of damage, particularly those beneath the surface or involving micro-cracking and delamination. Thirdly, factors such as the environmental conditions and the state of the specimen at the time of impact, including temperature and material degradation, can notably affect the appearance of damage.

Although advanced imaging and color profiling methods greatly aid in visually examining and evaluating impact damage in composite structures, acknowledging and addressing the intrinsic limitations in accurately depicting damage dimensions is essential for a comprehensive analysis.

A Scanning Electron Microscope (SEM) Quanta 650 was used to analyze a specimen that had undergone a Low-Velocity Impact (LVI) with an energy of 2.5J, intending to record the damage perimeter properly. The specimen was precisely cut with a diamond saw to reveal the impact-affected interior sections, allowing for high-resolution imaging that recorded the finer details of the damage.

**Fig. 6:**
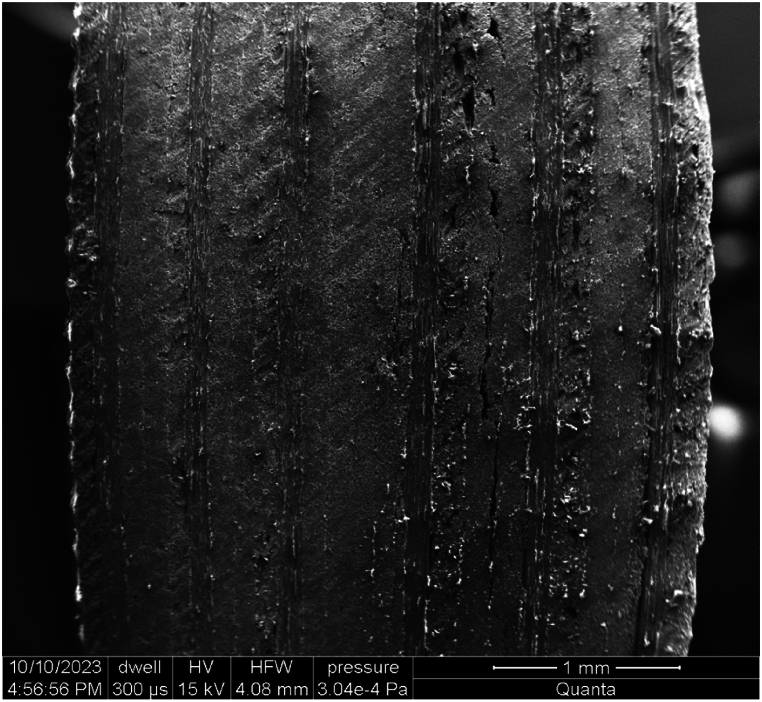
An SEM image of a sample after damaged by an LVI.

The scanning findings in Fig. 6 revealed damage between the 16th and 18th layers and between the 22nd and 23rd layers of the composite material. These findings validated the predicted results of a 2.5J LVI, revealing critical information about the specimen's structural integrity and damage processes.

### 2.2 Speckle patterns application

Speckling for DIC is an essential preparatory step that provides precise and reliable measurements by recording and analyzing surface images of a specimen while it deforms, allowing surface strains and displacements to be estimated. The quality and arrangement of the speckles are critical for the software's ability to monitor and correlate pictures effectively.

To ensure adept random dot distribution for DIC analysis, speckle patterns were applied to the specimens with high-contrast black and white spray paint technique. The speckle size was adjusted to match the cameras' resolution, with an average speckle diameter of 0.1 mm. After application, the samples are brushed to eliminate any stray particles that may have fallen during testing and interfered with the quality of reference points required for the DIC to work properly.

**Fig. 7:**
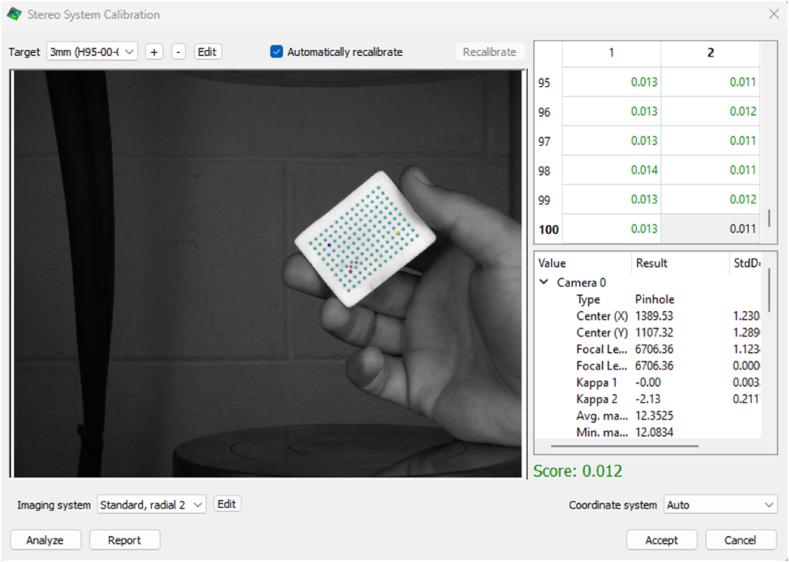
Calibration results using Vic-3D.

The next step is to take photographs of the 3 mm grid calibration tile with the Vic-Snap program, rotating and tilting it sideways to capture all angles. The Vic-3D software is launched after collecting at least 50 photos. The calibration photos option is then selected, and the photographs obtained are loaded. The caliber icon is chosen, instructing the program to extract points from pictures captured with both cameras and attempt to correlate them. In this case study, the calibration score was 0.012 (Fig. 7), which is below the criterion of 0.1. Otherwise, acquiring a new set of photos and repeating the calibration procedure is required before commencing the analysis.

A table with the calibration parameters and magnification is generated based on the calibration results, showcasing the coordinate system's location, focal length, and magnification parameters (Table 1).

**Table 1:** Camera calibration parameters and magnification.

Parameter	Camera 1	Camera 2
Center x [pixel]	1389.53	1391.86
Center y [pixel]	1107.32	1112
Focal length x [pixel]	6706.36	6711.74
Focal length y [pixel]	6706.36	6711.74
Skew	0	0
Kappa1	−0.00288777	−0.0058589
Kappa2	−2.1343	−1.62553
Kappa3	0	0
Average magnification [pixel/mm]	12.3525	12.2806
Minimum magnification [pixel/mm]	12.0834	12.0447
Maximum magnification [pixel/mm]	12.7584	12.6744

### 2.3 Evaluating CFRP samples under compression after impact (CAI)

The CAI test, initially conforming to ASTM D7137/D7137M standards with sample dimensions of 152.4 mm × 101.6 mm, was adjusted due to the limitations of the X-ray chamber. The adjustments involved a resizing process, bringing the dimensions of the samples down to 100 mm × 80 mm. This adjustment necessitated the need to aliterate the testing processes by compressing the impacted specimen in a custom-made fixture designed to accommodate the new dimensions of the samples for precise testing. This fixture, made from 7075 aluminum alloy, features a matte black finish to aid in Digital Image Correlation (DIC) analysis by reducing glare.

**Fig. 8:**
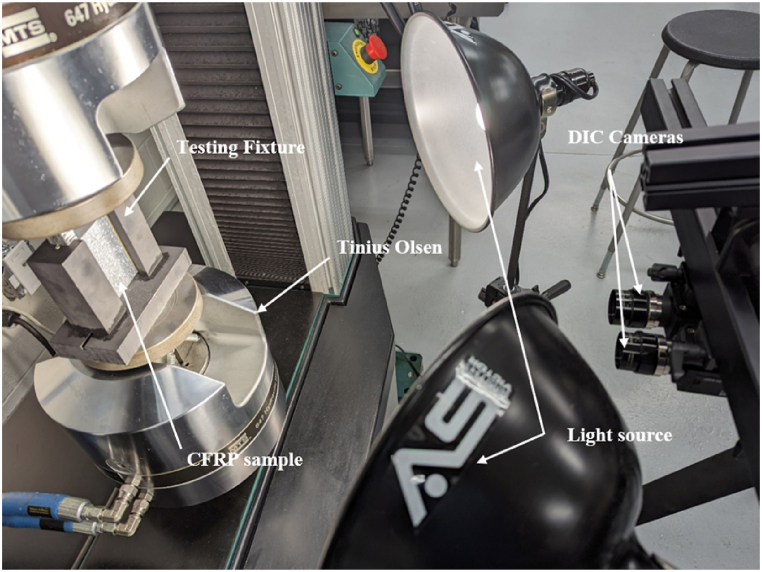
DIC setup facing a CAI testing fixture mounted on the Tinius Olsen testing machine.

The testing process includes mounting the fixture on a Tinius Olsen testing machine and using DIC to monitor the specimen's displacement, stresses, and strains for signs of delamination propagation (Fig. 8).

The loading protocol for the CAI test involves applying a compressive force at a gradual rate, set explicitly at 0.00208 % strain per second, translating to a displacement rate of 0.125 mm per minute. This controlled approach ensures that the loading process is precise and consistent, with the test designed to pause once a displacement of 0.75 mm is reached. This displacement threshold, determined from preliminary testing, is deemed optimal for observing the progression of delamination without reaching total specimen failure.

### 2.4 Assessment of displacement and strain in CAI testing

**Fig. 9:**
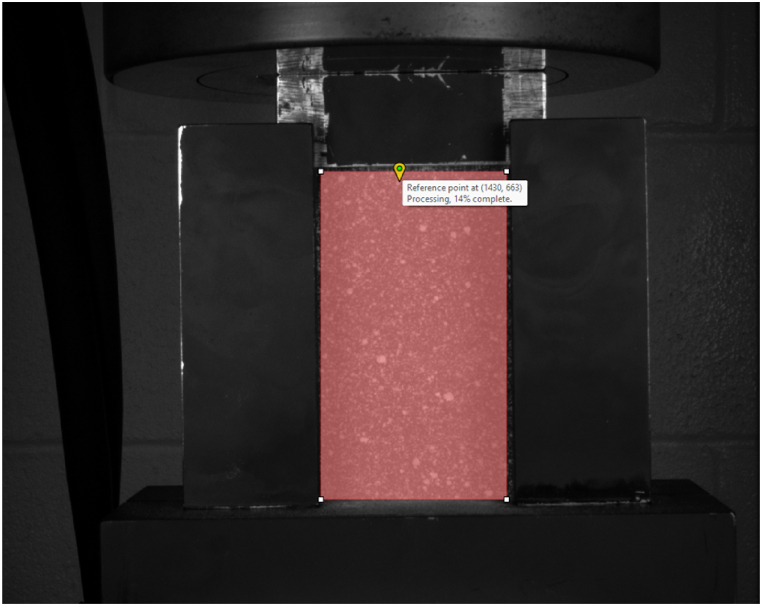
The entire specimen resolved for DIC with high-definition images.

The detailed analysis phase using Vic-3D software starts with selecting speckle images for a specific specimen and defining an Area of Interest (AoI) on these images. A reference point is also established to ensure consistent tracking across images captured by different cameras, aiding in the accurate autocorrelation of the speckle patterns (Fig. 9). Later, a subset and a step size are determined using the software's automated recommendation feature before running the analysis.

**Fig. 10:**
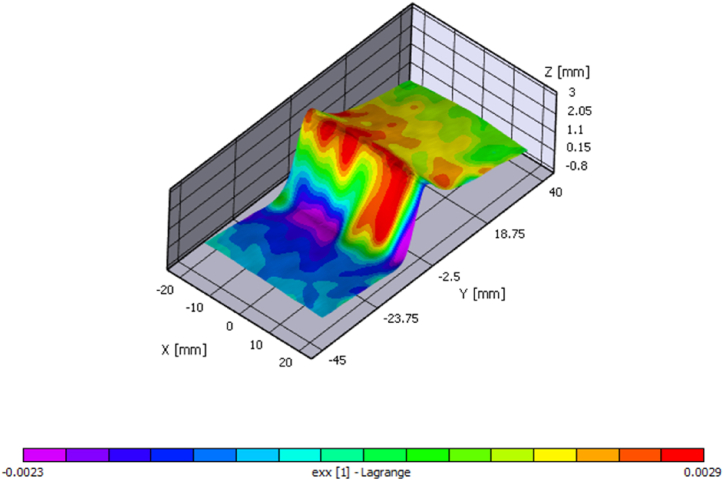
Strain mapping and 3-D visualization of out-of-plane displacement.

Following the completion of the Vic-3D analysis, the process transitions to strain computation, as seen in Fig. 10. Here, filter size and tensor type are chosen to tailor the calculations to the specific needs of the analysis. The subsequent step involves removing the rigid motion to distinguish between the specimen's overall movement and the deformation patterns of interest.

Finally, after eliminating the effects of rigid motion, the analysis culminates in exporting the data to MATLAB format, allowing for an integrated analysis by correlating the observed displacements and deformations with stress measurements obtained through the Tinius Olsen control software.

### 2.5 Data analysis and processing techniques

Integrating and synchronizing data from different sources is crucial for investigating delamination reinitiation in CFRP structures after Barely Visible Impact Damage (BVID). This integration is managed through a MATLAB code consolidating datasets from the VIC-3D system and the Tinius Olsen software, which records mechanical stress. The script compares each test's timestamps and combines them into a structured array.

### 2.6 Post-CAI damage examination and mapping

**Fig. 11:**
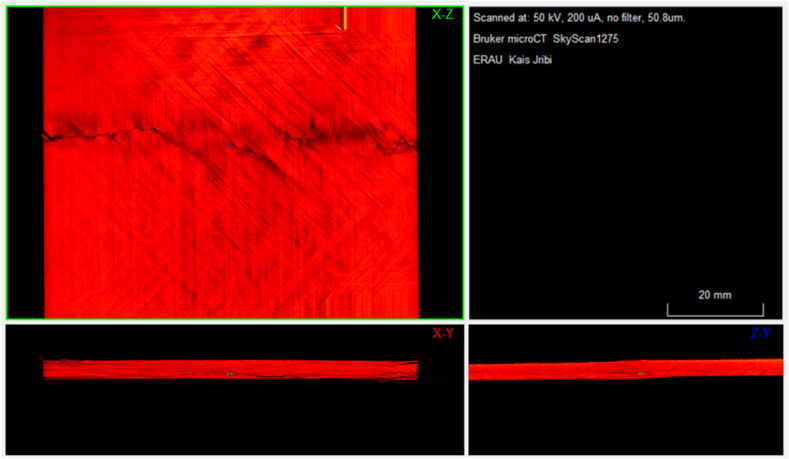
3D projection of the final damage topology from CT Scan.

**Fig. 12:**
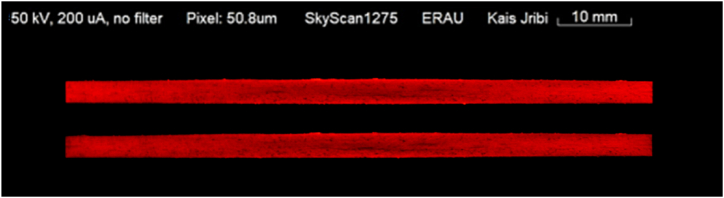
CT-Scan of a sample before and after CAI with 0.75 mm induced displacement (top: pre-CAI, bottom: post-CAI)), showing the increase in the delamination areas.

In the concluding phase of the experimental workflow, the damage incurred during the Compression After Impact (CAI) tests was evaluated through subsequent X-ray scanning of the specimens. Fig. 11 illustrates a specimen that was compressed until complete failure, revealing extensive damage and helping to understand the ultimate load-bearing capacity and failure modes of the composite material. In contrast, Fig. 12 depicts a controlled scenario where the sample was compressed up to a specific displacement of 0.75 mm, highlighting the onset of delamination reinitiation. By comparing the pre- and post-CAI scans, we can observe how the specimen's internal structure responds to compressive forces. Comparing the images helps identify the threshold at which delamination reinitiates, providing valuable data on the damage tolerance and critical thresholds for damage propagation in the material.

## 3 Results and discussion

### 3.1 Visualization of displacement patterns in BVID-affected CFRP

**Fig. 13:**
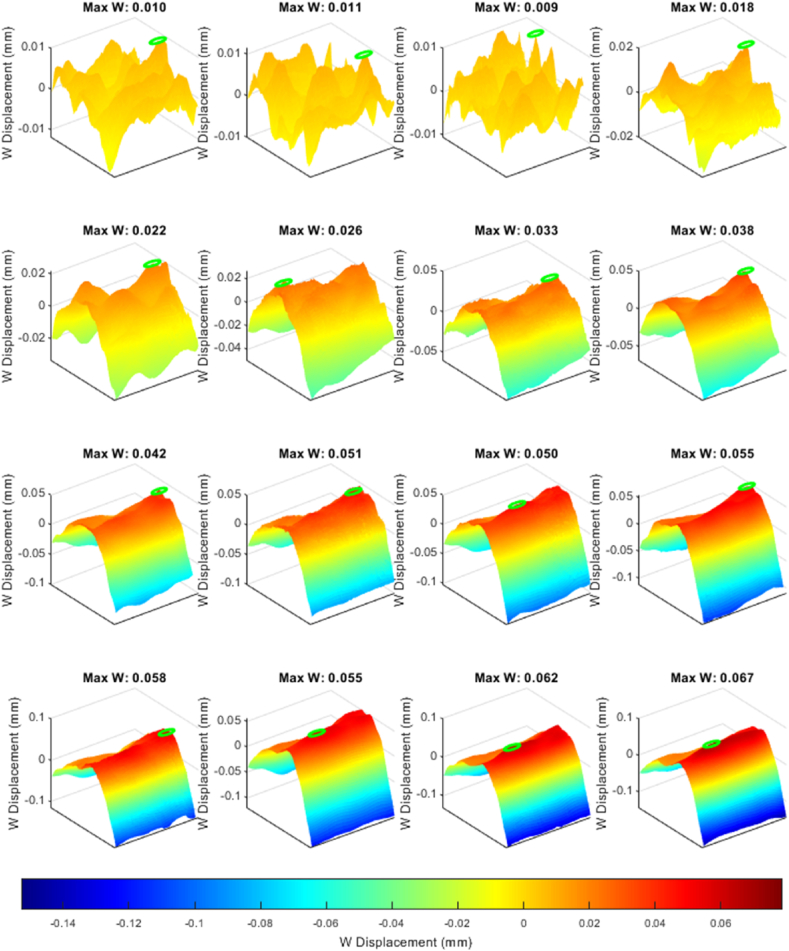
Identifying delamination reinitiation points via displacement in the out-of-plane direction.

A MATLAB script generates sixteen frames with uniform spacing from the collected dataset. The initial observations from the upper four frames in Fig. 13 display displacement values ranging from −0.01 to 0.01 mm, indicating minimal variance in displacement, which can be attributed to noise rather than indicative of actual material deformation. This early phase data, therefore, does not yield significant insights into the BVID behavior under a CAI.

As the dataset is further analyzed, a notable shift in the displacement pattern occurs, evolving into an inverted parabolic shape. This shift highlights a consistent pattern in the W (out-of-plane) displacement, marking the beginning stages of damage development within the CFRP samples. The small green circle in each map pinpoints the position with maximum out-of-plane displacement. Notably, a frame from the third row showing a W displacement of 0.05 mm is critical for pinpointing the BVID impact damage's location and defining the initial delamination boundary. After this point, the position of the maximum measured displacement remains in the same position. This frame effectively identifies the core area affected by the impact, with subsequent frames showing how the delamination expands, further delineating the damage site.

However, while the 3D images in Fig. 13 suggest the onset of damage propagation, interpreting these signs must be cautiously approached. The potential for data noise to mimic signs of further damage necessitates careful analysis to distinguish between genuine damage indicators and false positives.

**Fig. 14:**
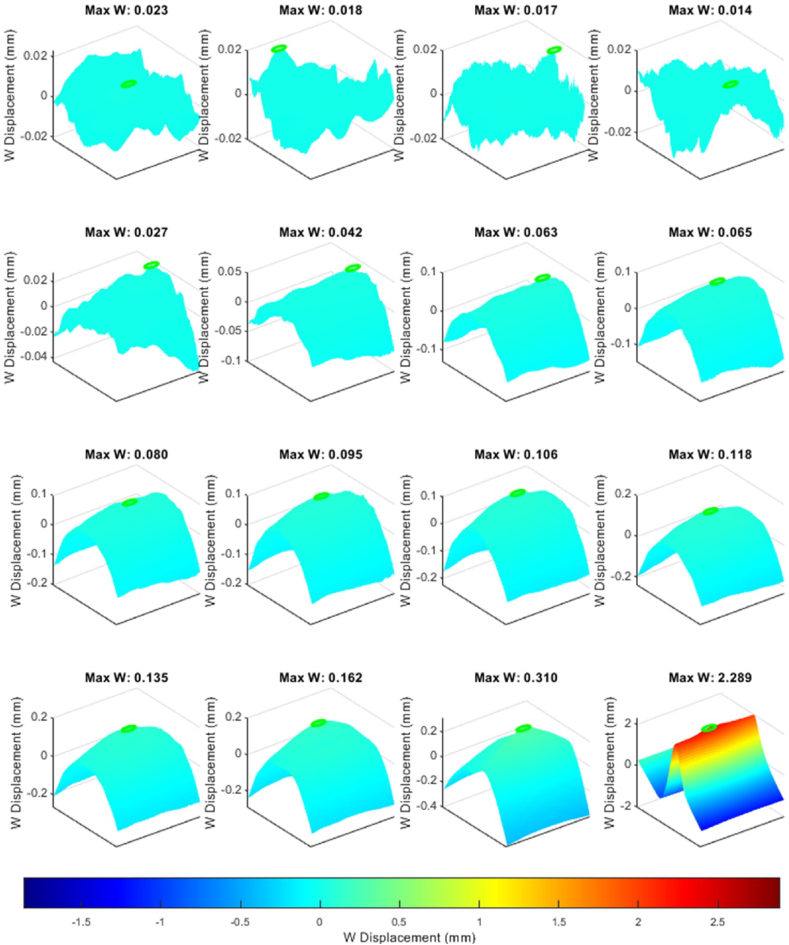
Evaluating CFRP response beyond 0.75 mm compressive threshold where the samples reached the total failure load causing a significant jump in the out-of-plane direction.

**Fig. 15:**
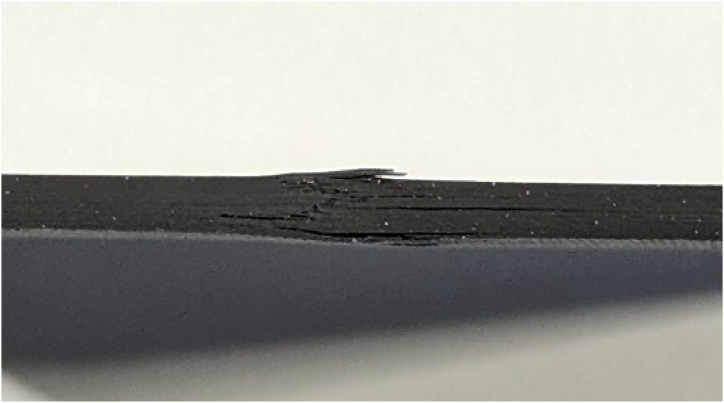
Structural failure in CFRP due to excessive compressive loads.

Fig. 14 illustrates a similar behavior trend, with the key difference being that the applied compressive load on the specimen exceeded the 0.75 mm displacement threshold set to detect the reinitiation of delamination, leading to the specimen's eventual failure, which can be observed in Fig. 15.

### 3.2 Stress shifts and impact energy correlation

**Fig. 16:**
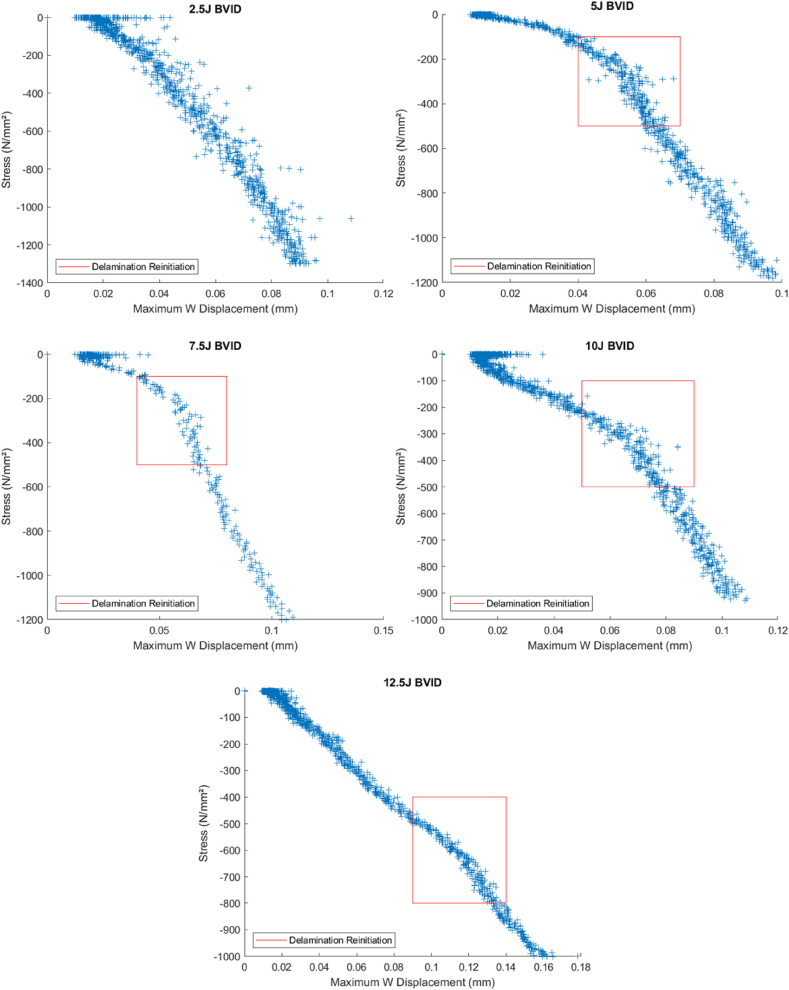
Peak displacement vs. stress at different BVID perimeters for ‘AS’.

The relationship between peak out-of-plane displacement and stress is one of the standard methods to identify delamination initiation from a BVID in composite materials. When analyzing the data, a notable shift in the stress values signals the reinitiation of delamination at the impact site. The shift can be easily observed in samples with BVID damage from impact energies starting from 5 J and up to 10 J, as seen in Fig. 16. This shift, highlighted by a red rectangle in the images for clarity, does not pinpoint an exact stress value but identifies a critical stress range where delamination is likely to reinitiate, consistent with the observation by Sun et al. Al [21].

Notably, damage from lower impact energies, specifically 2.5 J, is too minimal to be detected through stress-value variations. Similarly, damage resulting from higher impact energies, such as 12.5 J, becomes more difficult to detect due to the severity of the damage obscuring the stress shift. This limitation highlights the method's sensitivity range, emphasizing its applicability primarily to intermediate impact energies.

### 3.3 Integrating surface strain and calculated stress

An alternative approach involving the analysis of strain values at the surface is employed to detect the exact point of delamination initiation. This method leverages the detailed observation of surface strain patterns, allowing for a more precise identification of when delamination begins. By correlating these strain values with the stress data, it becomes possible to pinpoint the specific stress level at which the material's structural integrity is compromised, reducing the uncertainty of the onset of damage reinitiation. The measured $\varepsilon_{xx}$, $\varepsilon_{yy}$, and $\varepsilon_{xy}$ strains are plotted in the same chart to facilitate the onset visualization.

**Fig. 17:**
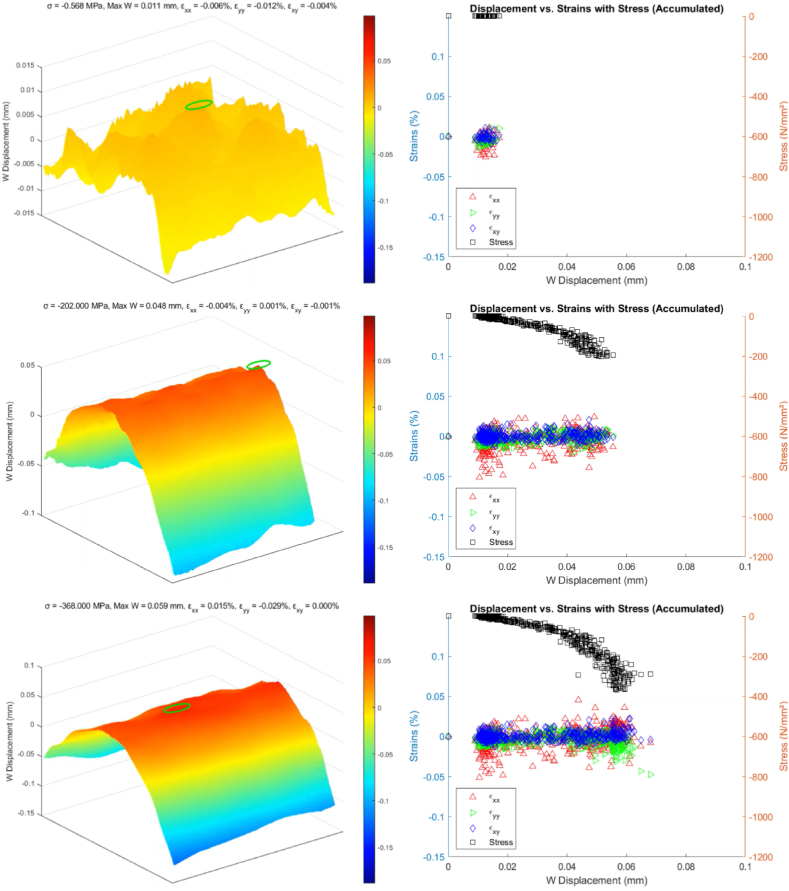

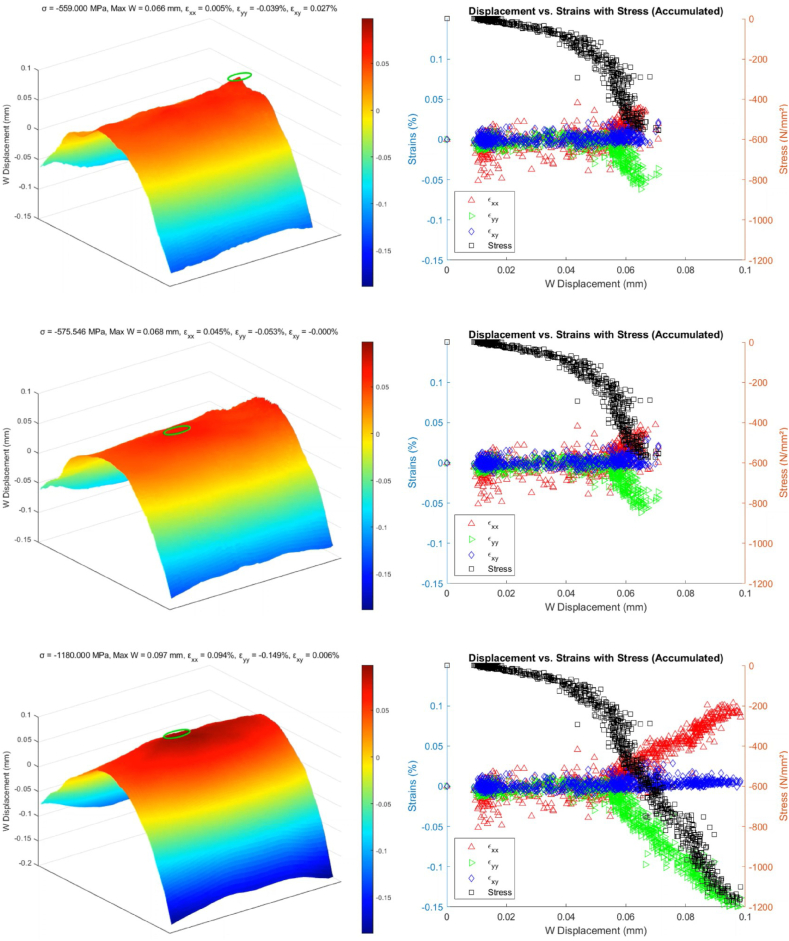
Step-by-step comparative analysis of CFRP response under CAI (5 J impact damage from ‘AS’)

Fig. 17 shows the evolution of the relationship between the measured displacements and strains as the load is applied, incorporating calculated stress values on a complementary axis. The saddle like plot is shown beside each chart to clearly show the point where the onset of failure is determined, as explained in section 3.1.

A clear trend is observed when the strains $\varepsilon_{xx}$ and $\varepsilon_{yy}$ begin to deviate from $\varepsilon_{xy}$ and each other. The point now is defining what deviation will be necessary to overcome the data uncertainty and define the unique point of the onset failure. This deviation is a critical indicator of the initiation of damage propagation from a BVID site, which can be further confirmed by the saddle charts, where the peak displacement is recorded at the BVID site. The figure also showcases the variation in the location of the highest W displacement caused by the noise in the data. The marker eventually marks the sample's midsection, where the BVID was introduced, and references the exact location as the displacement increases.

**Fig. 18:**
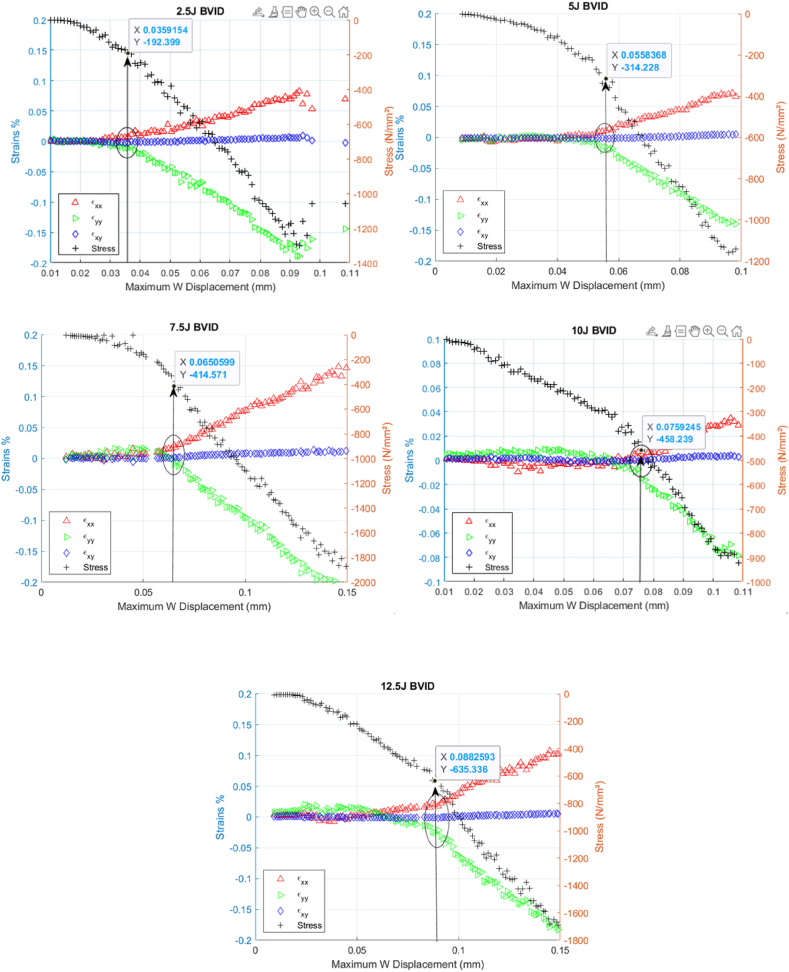
Comparative analysis of CFRP response under CAI testing for varying BVID damage perimeters from ‘as’.

Fig. 18 showcases the data from tests conducted at different impact energy levels and offers a detailed view of how these energies affect material behavior. For an impact of 2.5 J, the material begins to show strain divergence, “a point where the material's response to stress changes distinctly” at a displacement of 3.59 × 10^−2^ mm and under a stress of −192.4 MPa. When the impact energy is increased to 5J, this point of divergence shifts to a displacement of 5.58 × 10^−2^ mm and a stress of −314.2 MPa. At an even higher impact of 7.5 J, strain divergence is noted at a displacement of 6.51 × 10^−2^ mm, with the stress increasing to −414.6 MPa (see Table 2).

**Table 2:** Stress levels for delamination reinitiation.

Energy Levels	Stress (MPa)	Displacement (mm)
2.5 J	−192.4	3.59 × 10^−2^
5 J	−314.2	5.58 × 10^−2^
7.5 J	−414.6	6.51 × 10^−2^
10 J	−458.2	7.59 × 10^−2^
12.5 J	−635.3	8.83 × 10^−2^

The behavior continues moving to energy impacts, like those of 10J and 12.5J. At a 10J impact, the point of strain divergence is seen at a displacement of 7.59 × 10^−2^ mm with a stress of −458.2 MPa, and at a 12.5J impact, the displacement jumps to 8.83 × 10^−2^ mm, and the stress continues to rise to −635.3 MPa. Table 2 summarizes all the values measured from Fig. 18.

### 3.4 Onset of delamination reinitiation based on the delamination strain

The results show a clear trend based on the strains and the out-of-plane displacements at which delamination reinitiates in CFRP under varying impact energies. A new approach to determining the relationship is discussed in this segment.

**Fig. 19:**
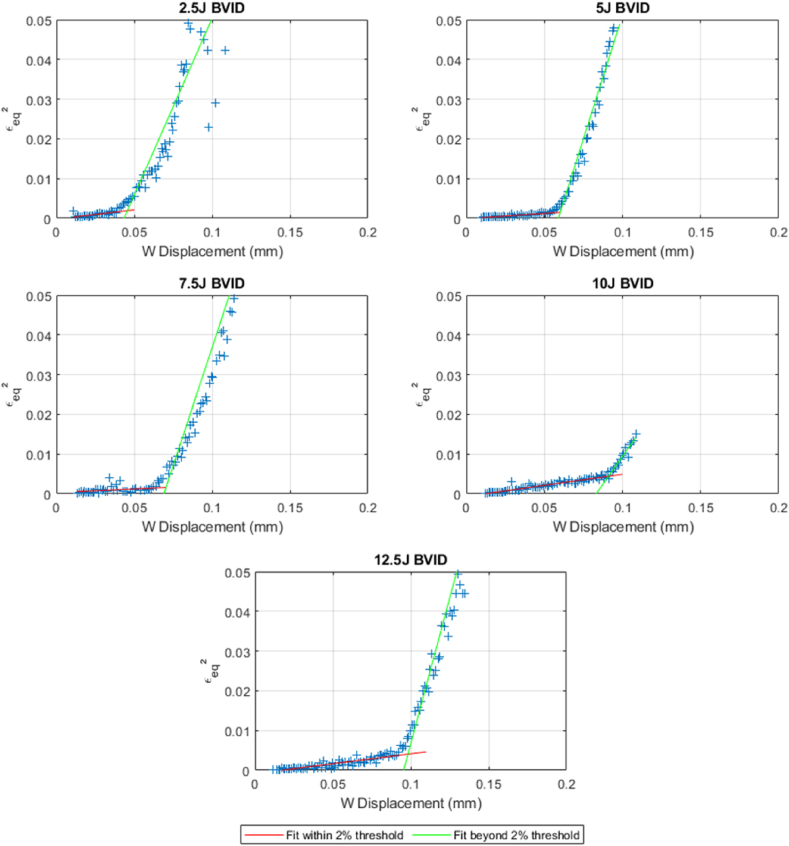
Peak displacement vs. The square of equivalent strain at different BVID perimeters for ‘as’.

This method integrates the full spectrum of global strains $\varepsilon_{xx}$, $\varepsilon_{yy}$, and $\varepsilon_{zz}$ by using the square of the equivalent strain $\varepsilon_{eqv.}$ as defined in the invariant of the deviatoric strain tensor $J_{2}^{\prime }$ [39] and is plotted against the out-of-plane displacement as seen in Fig. 19

(1) $$\varepsilon_{eqv.}^{2}=\frac{1}{2}\left[{\left(\varepsilon_{xx}-\varepsilon_{yy}\right)}^{2}+{\left(\varepsilon_{xx}-\varepsilon_{zz}\right)}^{2}+{\left(\varepsilon_{yy}-\varepsilon_{zz}\right)}^{2}\right]$$

Within the dataset of both laminate layup AS(45/90/-45/0/-45/0/-45/0/45/90/45/90)s and S(45/0/-45/90)3s, a perceivable trend similar to the one obtained from the analysis of a plate under buclkling appears where the data transition from following one linear trajectory to another. The intersection points of these linear fits have been identified and recorded, pinpointing the exact critical displacement ω at which delamination reinitiates across BVID from varying LVI energy levels.

**Fig. 20:**
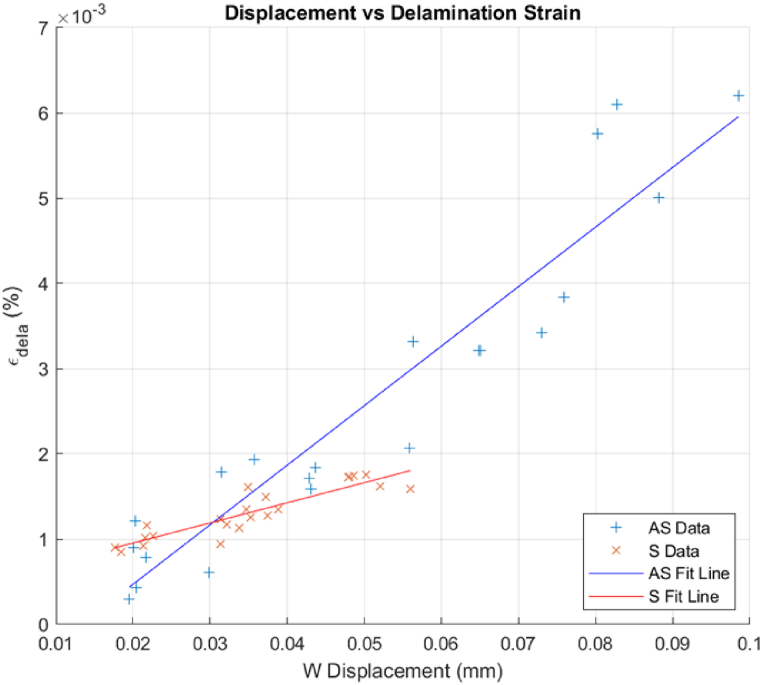
Displacement vs. Delamination strain for ‘as’ and ‘s’ layups.

The results shown in Fig. 19 are of great relevance because they clearly show unique points of critical loading condition for each initial damage level. Even being a stable condition, as in post-bucking of panel analysis, the points of inflection of the curves are very well defined here and are denoted by the intersection of two linear fits with an initial fit encompasses the square of equivalent strain values within 2 % shift whereas the second linear fit includes anything beyond the threshold. This measurement can be used to determine the level of loading condition for which the delamination would propagate. This approach is relevant in situations where the focus is on the reinitiation of damage propagation only without knowledge of the initial damage perimeters, particularly in BVIDs. Fig. 20 brings all the squares of equivalent strain values that correspond with the critical displacements, for all the performed experiments with all levels of initial damage.

In the comparative analysis of the onset of delamination reinitiation based on delamination strain, the data reveals distinct behaviors between the ‘AS’ and ‘S’ layups despite having the same thickness and material composition. The ‘AS’ layup has a steeper slope than the ‘S’. This difference implies that under the same impact and load conditions, ‘AS’ would experience a higher out-of-plane displacement before the delamination from higher impact loads reinitiates. The superior performance of ‘AS’ is attributed to the fiber orientation and layering sequence within the layup, which offers a better energy distribution upon impact and more effective resistance to delamination propagation [40].

A linear trendline was fitted for the ‘AS’ and ‘S’ layups, that can be used to approximate the reinitiation of the delamination based on the displacement and delamination strain extracted from the sample's surface with the help of the DIC.

## 4 Conclusion

Samples were manufactured according to aerospace standards and subjected to impact damage with different levels of beyond visual impact damage – BVID. Two different layup configurations were used, (45/90/-45/0/-45/0/-45/0/45/90/45/90)s denoted as ‘AS’ and a (45/0/-45/90)3s denoted as ‘S’. The internal damages were confirmed by inspection using X-Ray tomography. Compressive tests were conducted using the same specimens, to reinitiate the pre-induced impact delamination. Digital image correlation was used to map in-plain strains and to obtain the out-of-plane displacements in the specimen area interest during the experiments where the specimens were compressed to a prescribed longitudinal displacement. The sample holder was manufactured in compliance to ASTM D7137/D7137M standards, to perform compression experiments comparable to what has been done in previous research.

By mapping strains and displacements, we could establish a unique point that can be defined as the onset of crack reinitiation, as a function of strain field and out-of-plane displacement. The results corroborate the onset of failure previously found by researchers by the inflection of the stress curve as function of the out-of-plane displacement. We could superimpose the results for the strain maps and displacements with the stress curves, what showed a more accurately way to establish the onset of damage reinitiation. By fully mapping the out-of-plane displacement field, we could visualize the bulging surface, in a saddle-like curve, what confirmed the point where the loading became critical. Our experiments also showed that the ‘AS’ and ‘S’ layups have different resistance to damage reinitiation, however, both can be determined in the same method and with the same clarity of the onset point.

By mapping the strains, an energy derived term defined as $\varepsilon_{eqv.}$ could be calculated and plotted as a function of maximum the out-of-plane strain. These plots clearly define the inflection of the curve pinpointing the “moment” of damage reinitiation. This methodology allows for a swift and accurate inspection of structures subjected to compression after impact (CAI) tests. By leveraging this model, it can be expanded to efficiently predict and identify critical points of damage reinitiation, enabling more effective monitoring and maintenance of composite structures.

## CRediT authorship contribution statement

**Kais Jribi:** Writing – review & editing, Writing – original draft, Visualization, Software, Resources, Methodology, Investigation, Formal analysis, Conceptualization. **Boutros Azizi:** Validation, Resources, Investigation. **Alberto W. Mello:** Writing – review & editing, Validation, Supervision, Resources, Project administration, Methodology, Formal analysis, Conceptualization.

## Declaration of competing interest

The authors declare that they have no known competing financial interests or personal relationships that could have appeared to influence the work reported in this paper.

## References

[1] Reid S.R., Zhou G. (2000). Impact Behaviour of Fibre-Reinforced Composite Materials and Structures.

[2] Cook L., Boulic A., Harris D., Bellamy P., Irving P.E. (2013).

[3] Polimeno U., Meo M. (2009). Detecting barely visible impact damage detection on aircraft composites structures. Compos. Struct..

[4] Jribi K., Gosse J.H., Neill D.J., Mello A.W. (2023). ASME 2023 Aerosp. Struct. Struct. Dyn. Mater. Conf..

[5] Dubinskii S., Feygenbaum Y., Senik V., Metelkin E. (2019). A study of accidental impact scenarios for composite wing damage tolerance evaluation. Aeronaut. J..

[6] Dobyns A.L., Porter T.R. (1981). A study of the structural integrity of graphite composite structure subjected to low velocity impact. Polym. Eng. Sci..

[7] Fedulov B.N., Fedorenko A.N., Lomakin E.V. (2019). Evaluation of the residual strength of structures made of composite materials based on a Conservative distribution of damage parameters. IOP Conf. Ser. Mater. Sci. Eng..

[8] Poe C.C. (1996). Fatigue Fract.

[9] Fedulov B.N., Fedorenko A.N. (2020). Residual strength estimation of a laminated composite with barely visible impact damage based on topology optimization. Struct. Multidiscip. Optim..

[10] Davies G.A.O., Zhang X. (1995). Impact damage prediction in carbon composite structures. Int. J. Impact Eng..

[11] Zhang C., Zhang X., Duan Y., Xia Y., Ming Y., Zhu Y. (2021). Deformation resistance performance of carbon fiber-reinforced plastic machined by controlling drilling area temperature below the glass transition temperature. Materials.

[12] Liu D. (1988). Impact-induced delamination—a view of bending stiffness mismatching. J. Compos. Mater..

[13] Jribi K., Azizi B., Mello A.W. (2024). CRFP mechanical properties—stated values versus experimental data. J. Eng. Mater. Technol..

[14] Wang A., Slomiana M. (1982). Fracture Mechanics of Delamination. Initiation and Growth.

[15] Guinard S., Allix O., Guedradegeorges D., Vinet A. (2002). A 3D damage analysis of low-velocity impacts on laminated composites. Compos. Sci. Technol..

[16] Sarasini F., Tirillò J., Ferrante L., Valente M., Valente T., Lampani L., Gaudenzi P., Cioffi S., Iannace S., Sorrentino L. (2014). Drop-weight impact behaviour of woven hybrid basalt–carbon/epoxy composites. Composites, Part B.

[17] Goidescu C., Welemane H., Garnier C., Fazzini M., Brault R., Péronnet E., Mistou S. (2013). Damage investigation in CFRP composites using full-field measurement techniques: combination of digital image stereo-correlation, infrared thermography and X-ray tomography. Composites, Part B.

[18] Lau S.H., Chiu W.K.S., Garzon F., Chang H., Tkachuk A., Feser M., Yun W. (2009). Non invasive, multiscale 3D X-Ray characterization of porous functional composites and membranes, with resolution from MM to sub 50 NM. J. Phys. Conf. Ser..

[19] Lomov S.V., Ivanov D.S., Verpoest I., Zako M., Kurashiki T., Nakai H., Molimard J., Vautrin A. (2008). Full-field strain measurements for validation of meso-FE analysis of textile composites. Composer Part Appl. Sci. Manuf..

[20] Meola C., Carlomagno G.M. (2010). Impact damage in GFRP: new insights with infrared thermography. Composer Part Appl. Sci. Manuf..

[21] Sun X.C., Hallett S.R. (2018). Failure mechanisms and damage evolution of laminated composites under compression after impact (CAI): experimental and numerical study. Composer Part Appl. Sci. Manuf..

[22] Szebényi G., Hliva V. (2019). Detection of delamination in polymer composites by digital image correlation—experimental test. Polymers.

[23] Zhang L., Wang R., Liu W., Chen C., He X. (2012). Delamination growth behavior in carbon fiber reinforced plastic angle ply laminates under compressive fatigue loads. J. Reinforc. Plast. Compos..

[24] Yang Y. (2016). A numerical study of damage mechanisms in the CAI of laminated composites for aerospace applications. http://eprints.nottingham.ac.uk/33797/.

[25] Xu F., Liu W., Irving P.E. (2017).

[26] Wang K., Zhao L., Hong H., Zhang J., Hu N. (2020). An extended analytical model for predicting the compressive failure behaviors of composite laminate with an arbitrary elliptical delamination. Int. J. Solid Struct..

[27] Pascoe J.A., Alderliesten R.C., Benedictus R. (2013). Methods for the prediction of fatigue delamination growth in composites and adhesive bonds – a critical review. Eng. Fract. Mech..

[28] Justusson B., Liguore S., Schaefer J. (2019). Methods for composite structures service life extension – CALE demonstration. http://meetingdata.arctos-us.com/agenda/asip/2019/proceedings/presentations/P19303.pdf.

[29] Pan B., Qian K., Xie H., Asundi A. (2009). Two-dimensional digital image correlation for in-plane displacement and strain measurement: a review. Meas. Sci. Technol..

[30] Abbott T.B., Yuan F.-G. (2024). Subsurface impact damage imaging for composite structures using 3D digital image correlation. Struct. Health Monit..

[31] Sutton M.A., Matta F., Rizos D., Ghorbani R., Rajan S., Mollenhauer D.H., Schreier H.W., Lasprilla A.O. (2017). Recent progress in digital image correlation: background and developments since the 2013 W M murray lecture. Exp. Mech..

[32] Luo P.F., Chao Y.J., Sutton M.A., Peters W.H. (1993). Accurate measurement of three-dimensional deformations in deformable and rigid bodies using computer vision. Exp. Mech..

[33] Gao Y., Cheng T., Su Y., Xu X., Zhang Y., Zhang Q. (2015). High-efficiency and high-accuracy digital image correlation for three-dimensional measurement. Opt Laser. Eng..

[34] Chen B., Liu H., Pan B. (2020). Calibrating stereo-digital image correlation system using synthetic speckle-pattern calibration target. Meas. Sci. Technol..

[35] Malesa M., Malowany K., Pawlicki J., Kujawinska M., Skrzypczak P., Piekarczuk A., Lusa T., Zagorski A. (2016). Non-destructive testing of industrial structures with the use of multi-camera Digital Image Correlation method. Eng. Fail. Anal..

[36] Malowany K., Malesa M., Kowaluk T., Kujawinska M. (2017). Multi-camera digital image correlation method with distributed fields of view. Opt Laser. Eng..

[37] Kaware K.R., Kotambkar M.S. (2020). Effect of Impactor velocity and boundary condition on low velocity impact finite element modelling of CFRP composite laminates. IOP Conf. Ser. Mater. Sci. Eng..

[38] Sun X.C., Wisnom M.R., Hallett S.R. (2016). Interaction of inter- and intralaminar damage in scaled quasi-static indentation tests: Part 2 – numerical simulation. Compos. Struct..

[39] Hearn E.J. (1997). Mech. Mater, 2.

[40] Giasin K., Dhakal H.N., Featheroson C.A., Pimenov D.Y., Lupton C., Jiang C., Barouni A., Koklu U. (2021). Effect of fibre orientation on impact damage resistance of S2/FM94 glass fibre composites for aerospace applications: an experimental evaluation and numerical validation. Polymers.

